# The Effect of Saffron Supplementation on Blood Pressure in Adults: A Systematic Review and Dose-Response Meta-Analysis of Randomized Controlled Trials

**DOI:** 10.3390/nu13082736

**Published:** 2021-08-09

**Authors:** Leila Setayesh, Damoon Ashtary-Larky, Cain C. T. Clark, Mahnaz Rezaei Kelishadi, Pardis Khalili, Reza Bagheri, Omid Asbaghi, Katsuhiko Suzuki

**Affiliations:** 1 Department of Community Nutrition, School of Nutritional Sciences and Dietetics, Tehran University of Medical Sciences (TUMS), Tehran 14176-13151, Iran; setayesh.leila@yahoo.com; 2 Nutrition and Metabolic Diseases Research Center, Ahvaz Jundishapur University of Medical Sciences, Ahvaz 61357-15794, Iran; damoon_ashtary@yahoo.com; 3 Centre for Intelligent Healthcare, Coventry University, Coventry CV1 5FB, UK; ad0183@coventry.ac.uk; 4 Department of Community Nutrition, School of Nutrition and Food Science, Isfahan University of Medical Sciences, Isfahan 81746-73461, Iran; m.rezaei81@yahoo.com; 5 Department of Clinical Nutrition and Dietetics, Faculty of Nutrition and Food Technology, Shahid Beheshti University of Medical Sciences, Tehran 14167-53955, Iran; khalilipardis73@gmail.com; 6 Department of Exercise Physiology, University of Isfahan, Isfahan 81746-73441, Iran; will.fivb@yahoo.com; 7 Cancer Research Center, Shahid Beheshti University of Medical Sciences, Tehran 14167-53955, Iran; 8 Faculty of Sport Sciences, Waseda University, 2-579-15 Mikajima, Tokorozawa 359-1192, Japan

**Keywords:** saffron, blood pressure, systematic review, meta-analysis

## Abstract

Background: The favorable influences of saffron supplementation on metabolic diseases have previously been shown. We aimed to assess the effects of saffron supplementation on blood pressure in adults. Methods: A systematic search was performed in Scopus, Embase, and the Cochrane library databases to find randomized controlled trials (RCTs) related to the effect of saffron supplementation on blood pressure in adults up to March 2021. The primary search yielded 182 publications, of which eight RCTs were eligible. Results: Our results showed that saffron supplementation resulted in a significant decrease in systolic blood pressure (weighted mean difference (WMD): −0.65 mmHg; 95% CI: −1.12 to −0.18, *p* = 0.006) and diastolic blood pressure (DBP) (WMD: −1.23 mmHg; 95% CI: −1.64 to −0.81, *p* < 0.001). Moreover, saffron supplementation reduced DBP in a non-linear fashion, based on duration (r = −2.45, *p*-nonlinearity = 0.008). Conclusions: Saffron supplementation may significantly improve both systolic and diastolic blood pressure in adults. It should be noted that the hypotensive effects of saffron supplementation were small and may not reach clinical importance.

## 1. Introduction

High blood pressure, also known as hypertension (HTN), is one of the most severe and prevalent global diseases, estimated to be implicated in 9.4 million global deaths annually [1]. The outcomes of empirical studies have indicated a positive correlation between high blood pressure (BP) and the incidence of chronic diseases, such as cardiovascular diseases [2], myocardial infarction [3], and kidney dysfunction [4,5]. To mediate such issues, controlling BP is essential. Healthy dietary habits and exercise are more preferable than medication, mainly related to the contraindications and side effects of pharmacological intervention [6,7,8]. Thus, the use of alternative herbal-based remedies is widespread [9], particularly since they generally relieve symptoms and cause significantly fewer side effects [10].

Empirical research has demonstrated the positive effect of medicinal plants, particularly saffron, on lowering BP [11]. Indeed, saffron is abundant in crocin, picrocrocin, safranal, and crocetin [12], where such constituents are posited to confer positive effects on human health.

Saffron stigmas have long been used as a spice and a food coloring, concomitant to saffron’s practicality in food supplements [13,14]. Medicinally, saffron is known for its antimicrobial, antioxidant [15], liver protection [16], anti-inflammatory [17,18], nervous system protection [19], fat amelioration [20,21,22], anti-depressant [23,24], and anti-cancer [25] properties. The effective role of saffron as an antioxidant is mainly due to crocin-1 and crocin-2 [26]. Saffron includes approximately 30% crocin, 5–15% picrocrocin, and 2.5% fugacious compounds, although the exact make-up varies by genus [27]. The results of both animal and human research have demonstrated the positive effect of saffron on various diseases [28,29]. In contrast, the positive impact of saffron on some metabolic diseases, such as hypertension and dyslipidemia in murine models, has also been indicated [12,30,31]. Although the results of some human-based studies indicate a positive effect of saffron on blood pressure, there is a distinct lack of an overarching consensus. Therefore, we aimed to conduct a systematic review and meta-analysis to determine the influence of saffron supplementation on BP in adults.

## 2. Materials and Methods

### 2.1. Literature Search

The current study was conducted according to the PRISMA (preferred reporting items for systematic reviews and meta-analyses) guidelines [32]. Without any limitations on time and publication language, the following databases were used to identify suitable studies: PubMed, SCOPUS, EMBASE, Cochrane’s Library, and the ISI Web of Science. Search terms were related to saffron and its constituents (“saffron” OR “crocus” OR “crocin” OR “crocetin” “*Crocus sativus* Linn” OR “Safranal” OR “saffron”) AND (“Blood Pressure” OR “Hypertension” OR “systolic blood pressure” OR “diastolic blood pressure” OR “SBP” OR “DBP”). The search strategy identified articles published since journal inception, up to March 2021.

### 2.2. Study Selection

All of the articles were transformed into EndNote (version X7, for Windows, Thomson Reuters, Philadelphia, PA, USA) by a researcher (O.A.) who conducted a literature search. After the elimination of duplications, two independent authors (O.A. and P.K.) separately analyzed all titles and abstracts of the remaining articles, to ascertain whether these studies were eligible for our meta-analysis based on the inclusion criteria. Any dissimilar attitudes between the two investigators were settled by a panel discussion.

### 2.3. Inclusion Criteria

All of the studies needed to be RCTs, including crossover studies in adults (<18 years), in addition to the following criteria of a measured BP, and the use of any saffron component (e.g., whole saffron, standardized extracts, parts of saffron, or specific saffron-derived compounds). We also included interventions that used saffron combined with standard medications; however, combined interventions with other supplement ingredients were excluded. Finally, studies with observational and animal study designs, and acute interventions that were not in the form of randomized allocation or placebo-control groups, were excluded.

### 2.4. Data Extraction

The studies were independently screened and extracted by two authors, and a third author resolved disagreements. The following data were extracted using a standardized extraction form: first-named author, date, study design, the sample size in both intervention and control groups, total study period and follow-up, the characteristics of the target population, such as age, sex, body mass index (BMI), comorbidities, intervention features (including dose, type, and duration of exposure), adverse events, and BP, before and after the intervention.

### 2.5. Quality Assessment

The quality assessments were performed using the Cochrane Collaboration’s tool [33] by two authors (O.A. and S.M.) independently. Cochrane Collaboration’s tool includes various different domains: sequence generation, allocation concealment, blinding of participants and staff, blinding of the outcome assessment, missing outcome measures, selective outcome reporting, and other biases. For each item in the tool, the assessment of the risk of bias is in two parts. Each domain was categorized with a low, high, and unclear risk of bias. Based on Cochrane Collaboration’s tool, the quality of each publication was considered as good (low risk of bias for >2 domains), fair (low risk of bias for two domains), or weak (low risk of bias for <2 domains) [33].

### 2.6. Data Synthesis and Statistical Analysis

The mean ± standard deviation (SD) of change in BP was used to calculate the effect size within intervention and control groups, using a random-effects model [34]. BP was expressed as the weighted mean difference (WMD) and 95% confidence intervals (CIs). The I-square and Q tests were used to assess the heterogeneity among studies. Subgroup analysis, based on saffron dosage, duration and type of intervention, and baseline systolic blood pressure (SBP) and diastolic blood pressure (DBP), was conducted to detect potential sources of heterogeneity [35]. The standard formula was applied to convert all other statistics to mean ± SD, while Hozo et al.’s system was applied to convert median and 95% CIs to mean and SD [36]. The following formula was used to convert standard errors of the mean (SEM): SDs by S.E.M × √n (n is the number of participants in each group).

All units were converted to the most frequently used unit. All analyses were performed using the STATA version 12 (Stata Corporation, College Station, TX, USA), with statistical significance accepted, a priori, at *p* < 0.05.

## 3. Results

### 3.1. Study Selection

**Figure 1:**
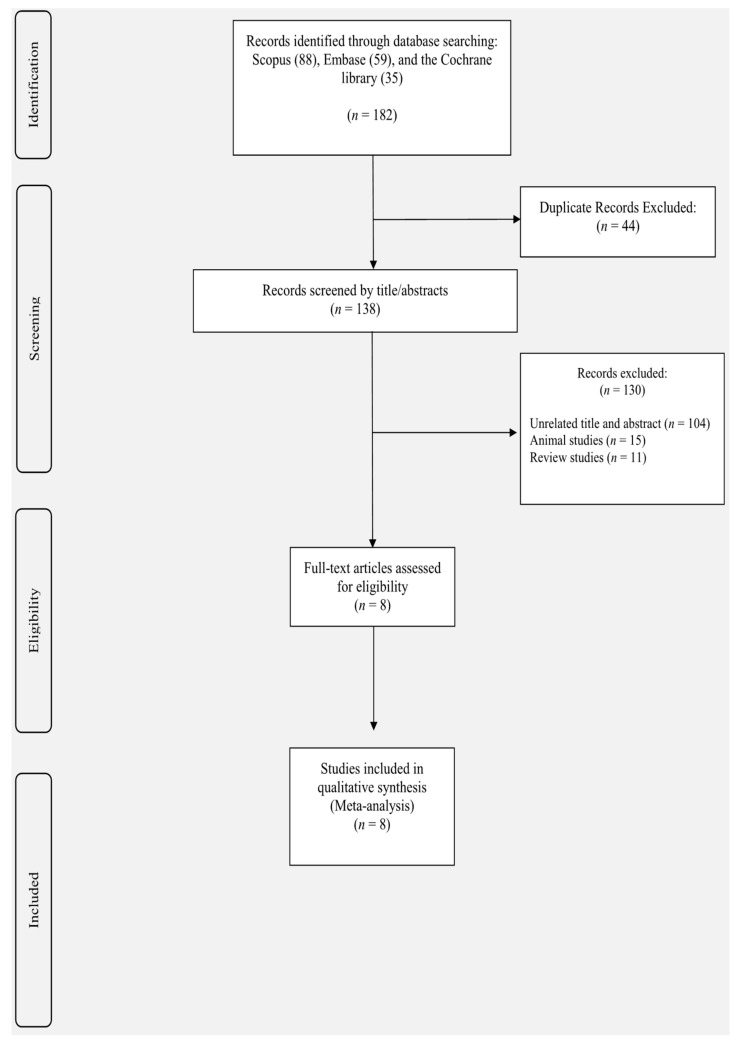
Flowchart of study selection for inclusion of studies.

Study selection was reported in Figure 1. The search strategy resulted in 182 studies through Scopus (*n* = 88), Embase (*n* = 59), and the Cochrane library (*n* = 35). Forty-four duplicated studies were excluded from our list, and 138 studies were screened to identify eligible studies. One-hundred and thirty studies were excluded, being animal-based (*n* = 15), review (*n* = 11), or unrelated studies (*n* = 104). Finally, after detailed scrutiny of the remaining articles, eight studies were included in our analysis.

### 3.2. Study Characteristics

Table 1 represents the characteristics of included articles, where all of them were randomized clinical trials (RCTs), published between 2008 and 2020, that were conducted in Iran [37,38,39,40,41,42,43,44]. The length of the intervention varied from 1 to 12 weeks, and the dosages of saffron supplementation were between 15 and 1000 mg. A cohort of 249 participants was collated in the intervention group, and 139 participants in the control group. The participants of these studies were classified as healthy volunteers [37], having type 2 diabetes [41,43,44], or with schizophrenia [38], asthma [42], or metabolic syndrome [39,40]. All of the studies enrolled both sexes, and only one was exclusively conducted on men [38]. Four studies used crocin [38,39,40], and the other seven studies used saffron. Two different studies that utilized multiple interventions (saffron and crocin) [38] and dosages (200 and 400 mg) [37] were considered as independent studies for the purposes of analysis.

### 3.3. The Effect of Saffron Supplementation on SBP

**Figure 2:**
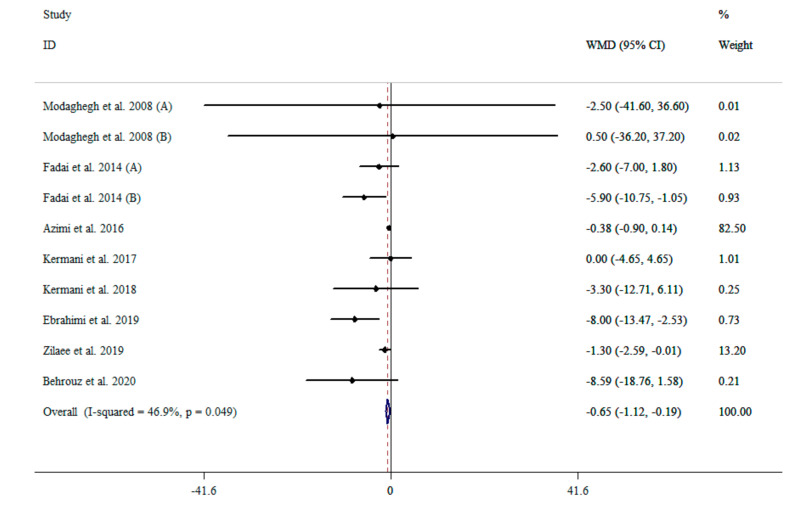
The effects of saffron supplementation on SBP.

The results of the pooled data from ten effect sizes [37,38,39,40,41,42,43,44] indicated that saffron supplementation reduced SBP (WMD: −0.65 mg/dL; 95% CI: −1.12 to −0.18, *p* = 0.006) compared with placebo, with moderate heterogeneity (I^2^ = 46.9%, *p* = 0.049) (Figure 2). Moreover, subgroup analysis showed that the saffron supplementation reduced SBP in all of the subgroups, except in those who had elevated SBP (>120 mmHg) (WMD: −0.47 mmHg; 95% CI: −0.98 to 0.03, *p* = 0.068) (Table 2).

### 3.4. The Effect of Saffron Supplementation on DBP

**Figure 3:**
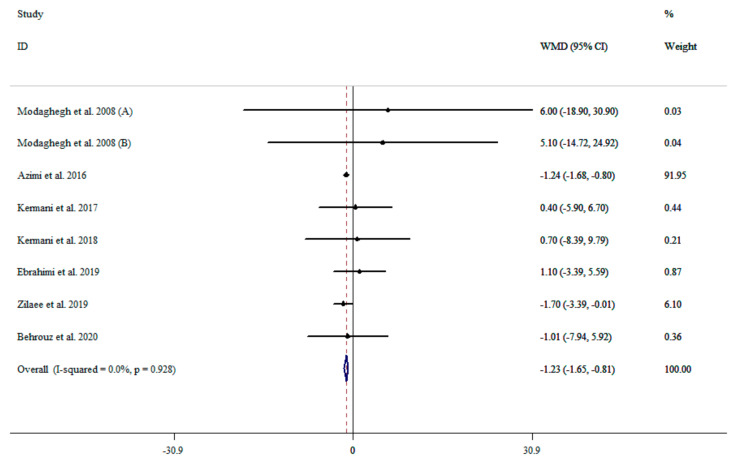
The effects of saffron supplementation on DBP.

Analysis of the eight effect sizes [37,39,40,41,42,43,44] indicated that saffron supplementation significantly reduced DBP (WMD: −1.23 mmHg; 95% CI: −1.64 to −0.81, *p* < 0.001) without significant heterogeneity (I^2^ = 0.0%, *p* = 0.928) (Figure 3). However, subgroup analysis showed that saffron supplementation could reduce DBP in participants with a baseline DBP ≥ 80 mmHg (WMD: −1.26 mmHg; 95% CI: −1.68 to −0.84, *p* < 0.001), in intervention durations less than 12 weeks (WMD: −1.25 mmHg; 95% CI: −1.68 to −0.83, *p* < 0.001), at dosages ≥100 mg per day (WMD: −1.23 mmHg; 95% CI: −1.65 to −0.81, *p* < 0.001), and interventions with crocin (WMD: −0.38 mmHg; 95% CI: −5.89 to 5.13, *p* < 0.001) (Table 2).

### 3.5. Non-Linear Dose-Response between the Doses and Duration of Saffron Supplementation and Blood Pressure

**Figure 4:**
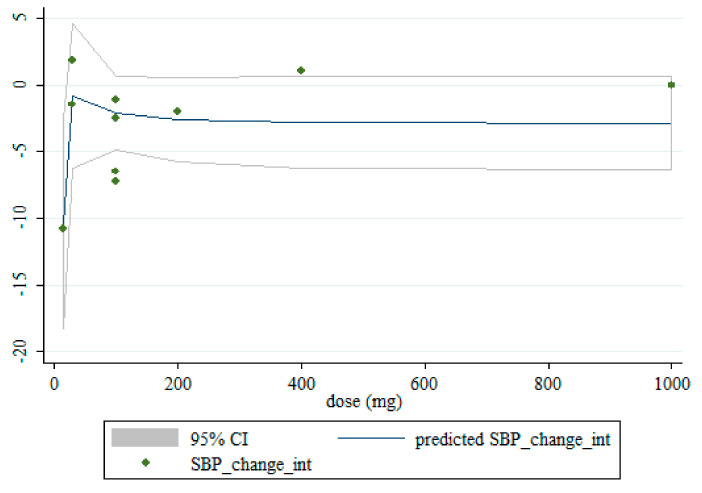
Non-linear dose-response relationships between saffron supplementation and absolute mean differences. Dose-response relationships between saffron dosage and absolute mean differences in SBP.

**Figure 5:**
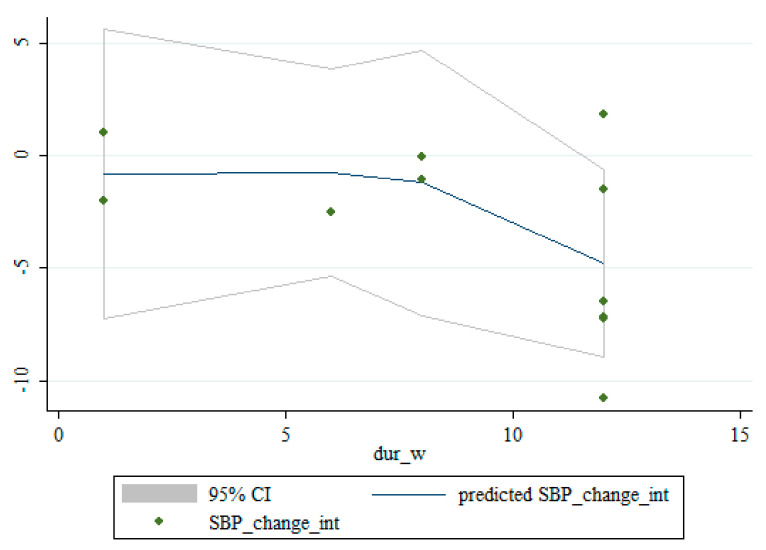
Non-linear dose-response relationships between saffron supplementation and absolute mean differences. Dose-response relationships between the duration of intervention and absolute mean differences in SBP.

**Figure 6:**
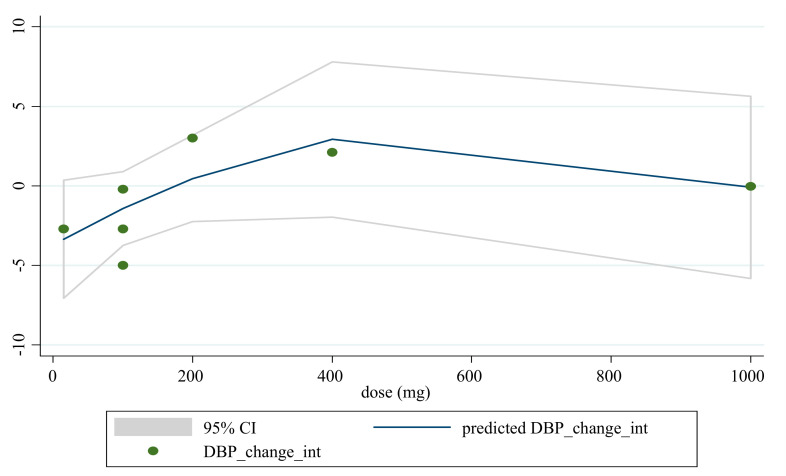
Non-linear dose-response relationships between saffron supplementation and absolute mean differences. Dose-response relationships between the saffron dosage and absolute mean differences in DBP.

**Figure 7:**
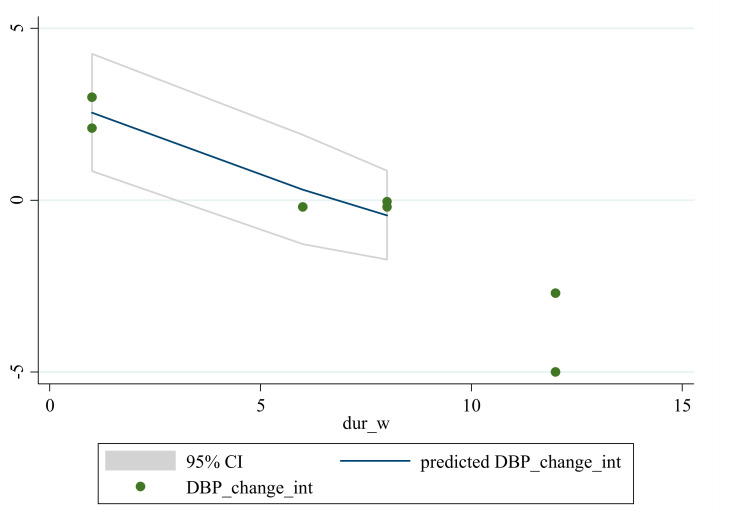
Non-linear dose-response relationships are shown between saffron supplementation and absolute mean differences. Dose-response relationships are shown between the duration of intervention and absolute mean differences in DBP.

Based on dose and duration, the dose-response analysis did not indicate any significant associations between saffron supplementation and changes in SBP. However, the dose-response analysis showed that saffron supplementation significantly altered DBP based on duration (*r* = −2.45, *p*-nonlinearity = 0.008) in a non-linear fashion (Figure 4, Figure 5, Figure 6 and Figure 7).

### 3.6. Publication Bias

**Figure 8:**
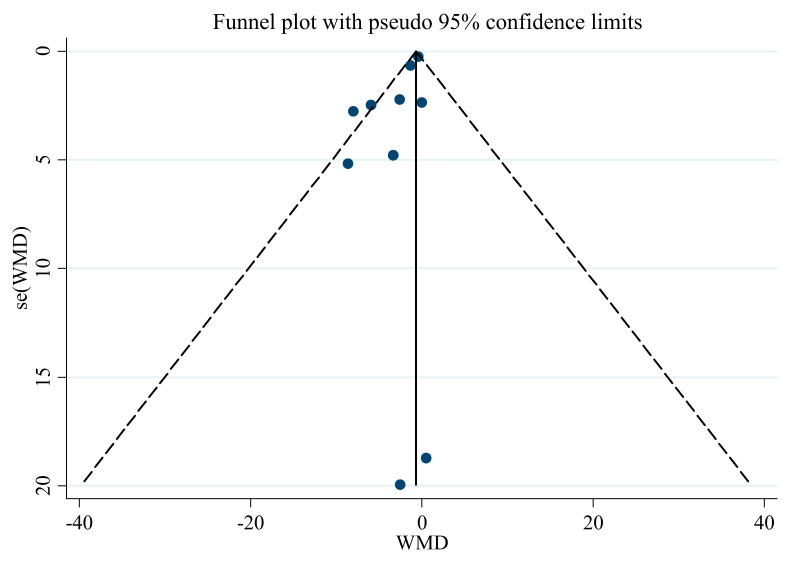
Funnel plot for the effect of saffron supplementation on SBP.

**Figure 9:**
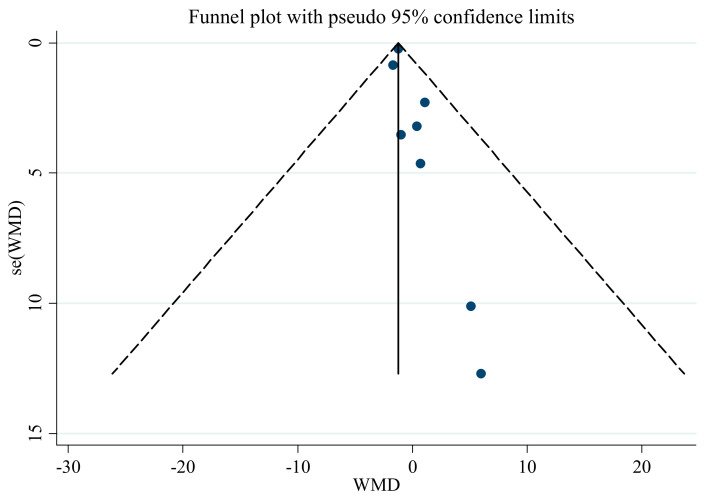
Funnel plot for the effect of saffron supplementation on DBP.

Publication bias assessment, using Egger’s regression test and an asymmetric funnel plot, showed no evidence of publication bias (Table 3) in the meta-analysis regarding the influence of saffron supplementation on SBP (*p* = 0.543). Nevertheless, there was significant publication bias for DBP (*p* < 0.001), with funnel plots indicating the same result (Figure 8 and Figure 9). Therefore, we conducted a trim and fill analysis for SBP. The results showed that when the publications reach 12, the publication bias changes significantly, but the overall results do not vary (WMD: −1.26 mmHg, CI: −1.67, −0.84; *p* < 0.001).

### 3.7. Sensitivity Analysis

Sensitivity analysis for SBP and DBP showed that the overall estimate was not affected by sequential omission of any study.

## 4. Discussion

Despite some reports in the literature indicating the positive influence of saffron supplementation on BP, there was a distinct lack of an overarching consensus. Thus, in this meta-analysis, we assessed the effects of saffron supplementation on BP in adults. Accordingly, we found that saffron supplementation was associated with a decrease in SBP and DBP when compared to a control group. Moreover, the hypotensive effects of saffron in decreasing DBP were greater with longer supplementation durations, while this dose-response was not evident for SBP.

It has been posited that some herbal medicines may be used for the prevention and treatment of chronic non-communicable diseases [46,47,48]. Indeed, some previous studies have reported that saffron supplementation and its components may reduce cardiovascular risk factors by improving the lipid profile [40,49,50], glycemic profile [50,51], and inflammation [51], as well as decreasing waist circumference [45,52]. Indeed, our results indicated improvements in BP variables, including SBP and DBP, and the hypotensive effects of saffron may be related to its components, including crocin, picrocrocin, safranal, and crocetin [12,53].

Our findings revealed that saffron supplementation has a small but significant effect on improving BP. Saffron may exert its antihypertensive effects through several mechanisms. For instance, animal-based studies have shown that saffron extract increases serum concentrations of nitric oxide (NO) [54,55]. Tang et al. indicated that crocetin significantly restored the endothelium-dependent relaxation of the thoracic aorta in hypercholesterolemic rabbits [56]. Moreover, Tang et al. also posited that these effects might be mediated by increasing endothelial NO synthase (eNOS) activity, leading to the elevation of NO production. In addition, saffron supplementation possesses numerous antioxidant properties, combats reactive oxygen species [57,58], and can enhance other antioxidant enzymes, such as catalase and superoxide dismutase [59,60], thereby protecting endothelial cells from oxidative damage and regulating BP. It has also been shown that saffron can act as a protective agent against cardiovascular disease by attenuating the NF-kappa B pathway [61,62]. Therefore, the anti-inflammatory effects of saffron may be another putative method by which hypotensive effects are exerted [63]. It has previously been indicated that crocetin can significantly down-regulate intercellular adhesion molecule-1 (ICAM-1) protein expression [64]. Indeed, endothelial dysfunction and an increased expression of ICAM-1 play roles in the initiation of the inflammatory process, which in turn has been reported to affect the renin-angiotensin system and contribute to hypertension [65]. Furthermore, in vitro and in vivo studies have demonstrated that saffron supplementation modulates adiponectin expression and secretion [66,67]. Evidence suggests that adiponectin has an influential role in regulating BP, and may reduce BP through anti-inflammatory, anti-atherogenic, and insulin sensitivity effects, concomitant to reversing salt-induced hypertension [68]. Overall, these findings indicate that saffron supplementation might be regarded as a potentially useful supplement for BP regulation. However, because of the small effect size, it should only be recommended with caution.

Despite the novelty of the results presented in this manuscript, our results should be interpreted while taking into consideration the following limitations. First, since all RCTs lasted less than three months, our analysis could not determine the long-term effects of saffron on BP. Although challenging to conduct, long-term research will help determine if the transient changes, or lack thereof, persist over time. Second, all studies were conducted in Iran, and this may represent a source of bias in our study. Indeed, this is out of the operational control of the study; however, we advocate that comparable studies should be conducted in a variety of geographical locations, so that this potential source of bias may be examined. Third, in most of the included studies, BP was reported as a secondary outcome, potentially harming the fidelity of measurements.

In the literature, saffron supplementation was associated with decreases in SBP and DBP when compared to a control group. Moreover, the hypotensive effects of saffron in reducing DBP but not SBP appeared to respond in a dose-response manner, whereby longer intervention durations were associated with a more significant decrease. However, it should be noted that the hypotensive properties of saffron supplementation were small and may not reach clinical importance. It has been mentioned that the minimal clinically important difference (MCID) is classified as clinically important, and is considered the smallest effect required to produce clinically important results [69]. The data for MCID regarding BP is limited; however, several studies have confirmed that the risk of hypertension, CHD, and stroke could be decreased whenever they saw reductions of ≥ 2 mmHg SBP and DBP [70,71,72]. Since WMD of the effects of saffron supplementation on both SBP and DBP is less than the MCID, we can consider that the beneficial effects of saffron on BP are not clinically significant. Further long-term and high-quality RCTs are needed to evaluate further and confirm the integrity of these findings. 

## Figures and Tables

**Table 1:** Characteristics of included studies.

Authors	Publication Year	Country	Study Design	Participant’s Sex	Sample Size		Participants	Duration (Week)	BMI		Age (Years)		Intervention/Control (Type and Dosage)		
					IG	CG			IG	CG	IG	CG	IG	Dose (mg)	CG
Modaghegh et al., 2008 (A) [37]	2008	IRAN	R/DB/PL	F/M	10	5	healthy volunteers	1	27.6	28.7	NR	NR	saffron	200	placebo
Modaghegh et al., 2008 (B) [37]	2008	IRAN	R/DB/PL	F/M	10	5	healthy volunteers	1	28.7	28.7	NR	NR	saffron	400	placebo
Fadai et al., 2014 (A) [38]	2014	IRAN	R/TB/PL	M	20	21	patients with schizophrenia	12	49.3 ± 7.1	48.1 ± 6.1	NR	NR	saffron	30	placebo
Fadai et al., 2014 (B) [38]	2014	IRAN	R/TB/PL	M	20	21	patients with schizophrenia	12	48.1 ± 7.7	48.1 ± 6.1	NR	NR	crocin	30	placebo
Kermani et al., 2017 [39]	2017	IRAN	R/DB/PL	F/M	22	22	metabolic syndrome	12	43.64 ± 11.17	42.59 ± 8.44	31.02 ± 5.45	30.48 ± 6.26	saffron	100	placebo
Kermani et al., 2017 [40]	2018	IRAN	R/DB/PL	F/M	24	24	metabolic syndrome	6	53.8 ± 9.2	50.9 ± 8.8	29.9 ± 3.9	29.8 ± 5.3	crocin	100	placebo
Ebrahimi et al., 2019 [45]	2019	IRAN	R/DB/PL	F/M	40	40	type 2 diabetic patients	12	55.2 ± 7.3	53 ± 10.6	29.3 ± 4.9	30.5 ± 4.7	saffron	100	placebo
Zilaee et al., 2019 [42]	2019	IRAN	R/DB/PL	F/M	38	38	patients with mild and moderate persistent allergic asthma	8	41.27 ± 9.77	40.77 ± 10.07	26.84	26.84	saffron	100	placebo
Behrouz et al., 2020 [43]	2020	IRAN	R/DB/PL	F/M	23	22	type 2 diabetic patients	12	57.08 ± 7.41	59.86 ± 9.46	30.64 ± 4.79	30.85 ± 3.19	crocin	15	placebo
Azimi et al., 2016 [44]	2016	IRAN	R/SB/PL	F/M	42	39	type 2 diabetic patients	8	57.02 ± 1.0	53.64 ± 1.3	28.86 ± 0.2	28.40 ± 0.2	saffron	1000	placebo

Abbreviations: R, randomized; DB, double-blind; SB, single-blind; PL, placebo; M, male; F, female; BMI, body mass index; IG, intervention group; CG, control group; NR, not reported.

**Table 2:** The effects of saffron supplementation on blood pressure.

	NO	WMD (95%CI)	*p* Value	P Heterogeneity	I^2^ (%)
Effect of saffron supplementation on SBP					
Overall effect	10	−0.65 (−1.12, −0.18)	**0.006**	0.049	46.9%
Baseline SBP (mmHg)					
<120	6	−1.57 (−2.73, −0.41)	**0.008**	0.565	0.0%
≥120	4	−0.47 (−0.98, 0.03)	0.068	0.017	70.6%
Duration					
<12	5	−0.51 (−0.99, −0.03)	**0.035**	0.729	0.0%
≥12	5	−4.00 (−6.34, −1.67)	**0.001**	0.155	40.0%
Intervention dose (mg)					
≥100	7	−0.56 (−1.03, −0.09)	**0.019**	0.160	35.1%
<100	3	−4.50 (−7.61, −1.40)	**0.004**	0.436	0.0%
Intervention type					
Saffron	7	−0.58 (−1.05, −0.11)	**0.016**	0.136	38.4%
Crocin	3	−5.84 (−9.81, −1.87)	**0.004**	0.755	0.0%
Effect of saffron supplementation on DBP					
Overall effect	8	−1.23 (−1.64, −0.81)	**<0.001**	0.928	0.0%
Baseline DBP (mmHg)					
<80	5	0.66 (−2.50, 3.83)	0.681	0.959	0.0%
≥80	3	−1.26 (−1.68, −0.84)	**<0.001**	0.800	0.0%
Duration					
<12	5	−1.25 (−1.68, −0.83)	**<0.001**	0.883	0.0%
≥12	3	0.45(−2.77, 3.69)	0.782	0.882	0.0%
Intervention dose (mg)					
≥100	7	−1.23 (−1.65, −0.81)	**<0.001**	0.871	0.0%
<100	1	−1.01 (−7.94, 5.92)	0.775	-	-
Intervention type					
Saffron	6	−1.23 (−1.65, −0.81)	**<0.001**	0.806	0.0%
Crocin	2	−0.38 (−5.89, 5.13)	0.892	0.769	0.0%

Abbreviations: SBP, systolic blood pressure; DBP, diastolic blood pressure.

**Table 3:** Risk of bias assessment of the studies included in this meta-analysis.

Study (Year)	Random Sequence Generation	Allocation Concealment	Selective Outcome Reporting	Other Sources of Bias	Blinding of Participants Personnel	Blinding of Outcome Assessors	Incomplete Outcome Data
Modaghegh et al., 2008 [37]	L	U	L	H	L	U	L
Fadai et al., 2014 [38]	L	U	H	H	L	L	L
Azimi et al., 2016 [44]	L	U	L	L	H	H	L
Kermani et al., 2017 [39]	L	U	L	H	L	U	L
Kermani et al., 2018 [40]	L	U	L	H	L	U	L
Ebrahimi et al., 2019 [45]	L	U	L	L	L	U	L
Zilaee et al., 2019 [42]	L	U	L	L	L	U	L
Behrouz et al., 2020 [43]	L	U	L	L	L	U	L

Abbreviations: U; unclear risk of bias, L; low risk of bias, H; high risk of bias.
